# Neomodified Koyanagi technique for severe hypospadias with one‐stage sealed Y‐shaped penis foreskin vascular protection surgery

**DOI:** 10.1002/ccr3.5575

**Published:** 2022-03-17

**Authors:** Jiancheng Zu, Yifu Chen, Yu Liu, Tianqu He, Yanling Wang, Yuee Zu, Xiangwen Peng

**Affiliations:** ^1^ 37046 Hunan Children's Hospital Changsha City China; ^2^ Changsha Hospital for Maternal and Child Health Care of Hunan Normal University Changsha City China

**Keywords:** Koyanagi technique, proximal hypospadias, surgery, Y‐shaped penis

## Abstract

Proximal hypospadias defects represent the most challenging aspect of maintaining blood supply to the flap, which eventually leads to a high rate of complications. We modified a sealed Y‐shaped penis foreskin vascular protection technique, which can repair the urethra in a single stage. The inner plate of the foreskin was cut along the coronal sulcus, and both sides of the urethral plate were cut as deep as Buck's fascia. The "Y"‐shaped foreskin flaps on both sides of the mouth that are continuous with the urethral plate were sutured to form a new urethral skin tube. The urethral skin tube was turned to the ventral side, and the foreskin was reshaped and sutured. A total of 89 children had their urinary catheters removed 4 weeks after the operation. All children were evaluated at least once a year for 3 consecutive years. There were 11 patients with urine leakage that occurred after the operation. These children, diagnosed with urine leakage, underwent successful repair after the leakage occurred. There were no urethral strictures after the operation. The one‐time success rate of this operation was 87.6% (78/89), and the incidence of urethral fistula was 12.6% (11/89). The results showed that sealed Y‐shaped penis foreskin vascular protection surgery was safer and had a higher operation rate than the traditional hypospadias repair technique. Modifying Koyanagi repair by our improved Koyanagi hypospadias repair is an excellent technique with relatively low complication rates.

## Graphical abstract

We designed a closed Y‐shaped flap repair. In this operation, we used the Y‐shaped flap to synthesize a new urethra. The unique incision to protect the vascular system of the Y‐shaped flap enables one‐time repair of hypospadias with complete repair of penile curvature with low complication rate.

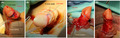

## 1 INTRODUCTION

Since the Koyanagi technique development, it has received much attention for its logical flap design.^1^, ^2^ Many modifications in the Koyanagi technique have been introduced because of its high complication rate.^3^, ^4^, ^5^, ^6^ However, the complication rate has not decreased much. We believe that the high complication rate is mainly due to interruption of the blood supply to the flap. Here, we developed a modified Koyanagi technique with improved blood supply to the flap for single‐stage repair of severe hypospadias, resulting in greatly reduced complications. Similar to the Koyanagi technique, the urethral plate is reconstructed with the foreskin, but then a sealed Y‐shaped incision is made in the original and reconstructed urethral plate. Unlike the Koyanagi technique of incising the foreskin, the modified technique only incises the superficial fascia layer, and the vascular system under the superficial fascia layer is protected. In this way, the blood supply of the flap is complete, and surgical failure caused by avascular necrosis of the flap is reduced. At the same time, all penile curvatures are corrected due to our use of an extended urethra.

## 2 OPERATIVE TECHNIQUE

Preoperative examination included chromosome, scrotum, groin, kidney, ureter, bladder, heart color ultrasound, liver and kidney function, and electrocardiogram studies. The vulvar area was washed with running water 12 h before surgery. Fasting was performed 6–8 h preoperatively, and cefuroxime was administered 30 minutes preoperatively. With the patient in the supine position, 0.05% complexed iodine was used to disinfect the skin, and 0.05% complexed iodine was used to disinfect the foreskin and urethral opening. Next, an F8 catheter was smoothly inserted into the bladder and left in, and 0.05% complexed iodine was again used to disinfect the foreskin. No. 1 silk thread was used to suture the dorsal side of the glans as a traction line.

**FIGURE 1:**
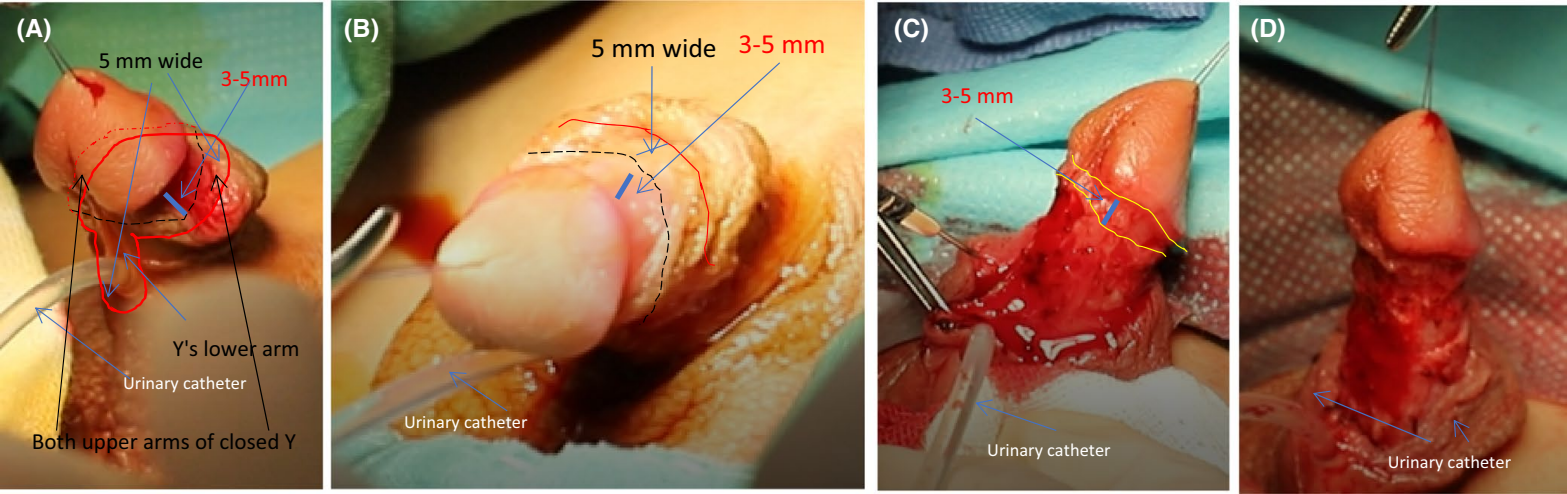
Circumcise the foreskin and separate the foreskin from the corpus cavernosum. A‐B: Use a laser knife to circumcise the circumcision (cut off the deep fascia layer) along the dotted line, leaving the foreskin on the glans side (3–5 mm). C‐D: The foreskin is separated from the corpus cavernosum albuginea along the corpus cavernosum. (The dotted line represents the circumcision route, and the solid red line represents the incision line in step 2, which creates a closed Y‐shaped flap)

**FIGURE 2:**
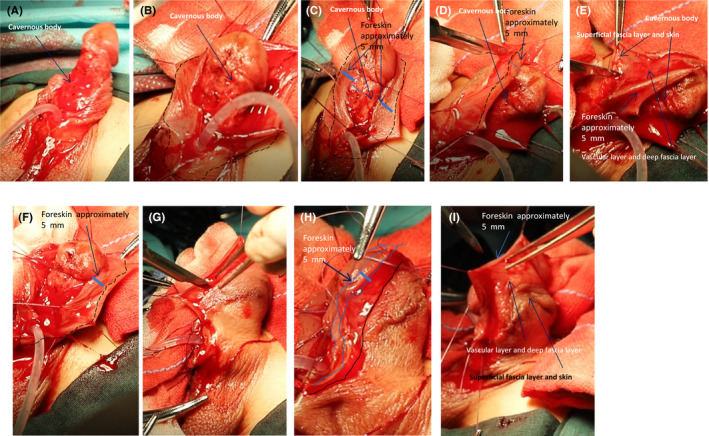
Schematic images illustration steps of surgery. (A‐B) Circumcision of the foreskin. (C‐E) Construction of a Y‐shaped flap. (F‐G) Suture of the new urethra. (H‐I) Location of the urethral opening and urethra. (J‐K) Location and suture of the coronal sulcus. (L) Complete suture

**FIGURE 3:**
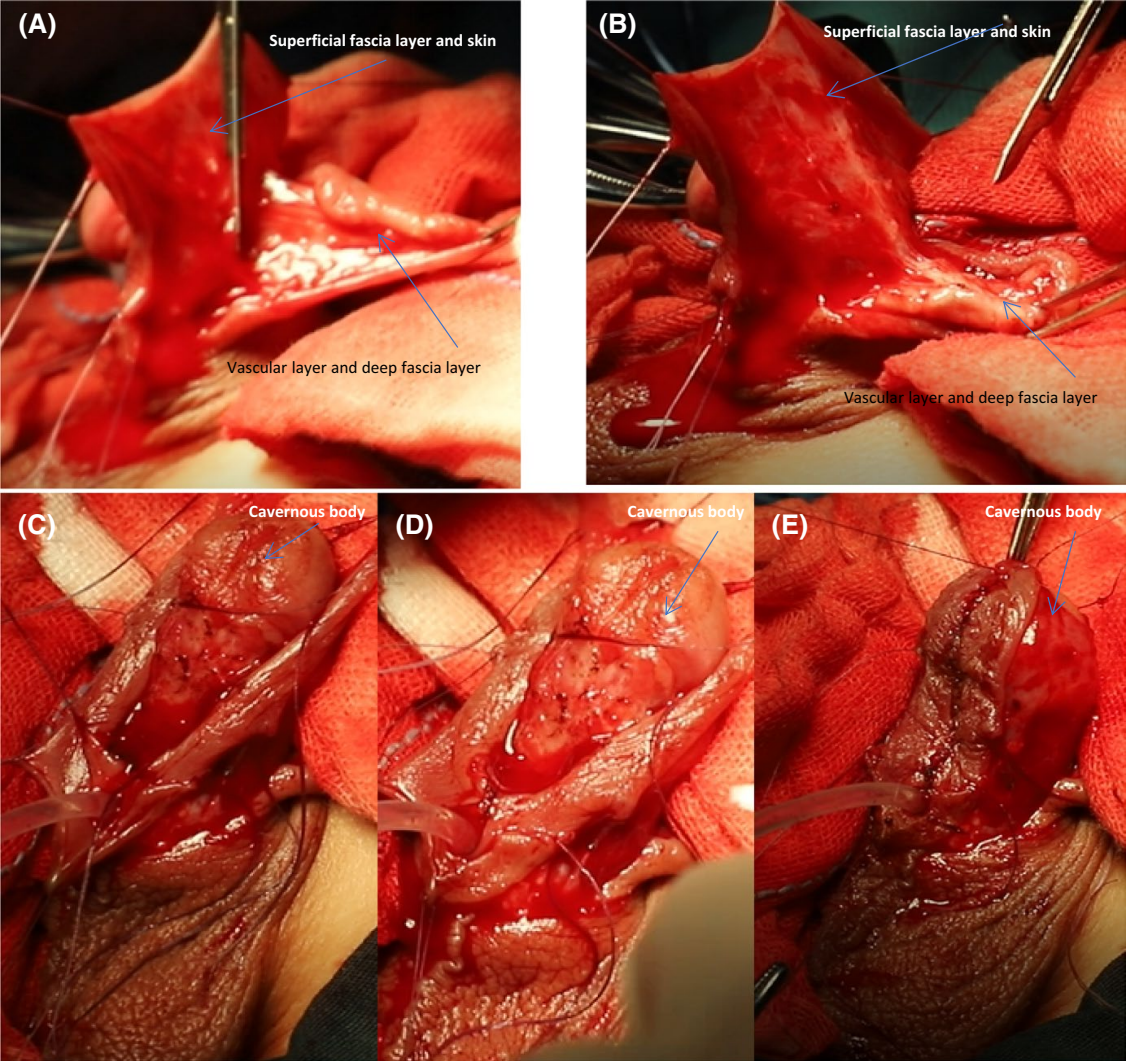
Create a 5‐mm‐wide, sealed, Y‐shaped skin flap. (A‐E) Cut the skin on the left to form the side of the Y. (F‐H) Cut the skin on the right to form the other side of the Y. (I) Cut the skin on the top to form the top side of the Y

**FIGURE 4:**
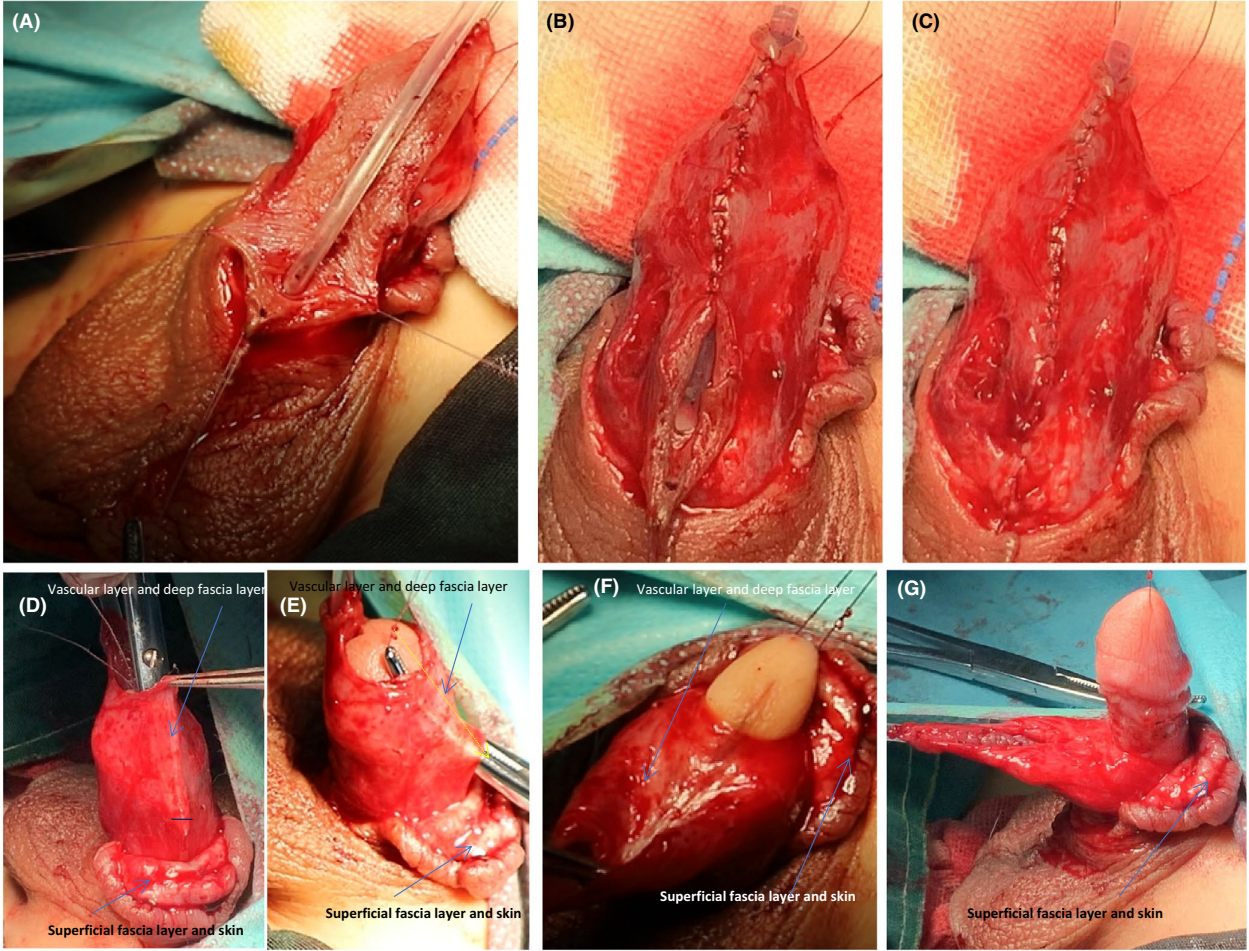
Another patient had distal hypospadias before and after surgery. (A) Preoperative photo of a patient with distal hypospadias and schematic diagram of the Y‐shaped flap tangent. The blue line represents the circumcision line, and the yellow line represents the tangent to the superficial fascia layer in step 2. (B‐C) Picture of the patient after recovery. (D) After recovery, the patient had an erection that did not bend

**FIGURE 5:**
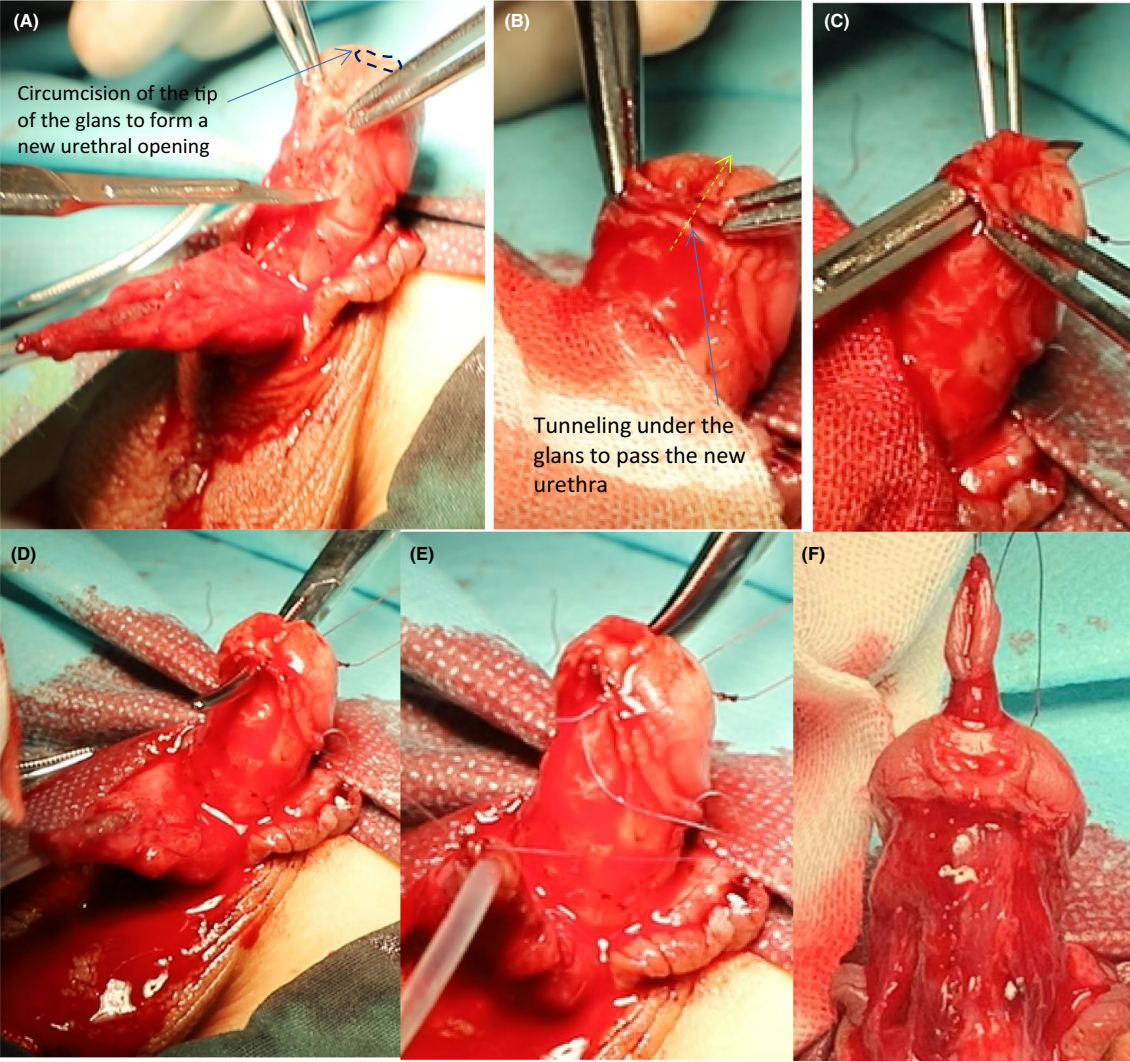
Layer the foreskin and suture the new urethral plate. (A‐B) Divide the dorsal foreskin into two layers. (C‐E) Suture the inside of the flap to form a new urethral plate

**FIGURE 6:**
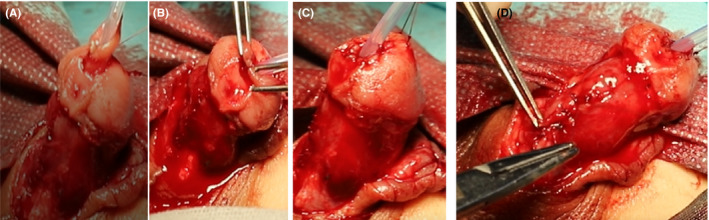
Close and correct the new urethra. (A‐C) The outer side of the new urethral plate is sutured correspondingly and closed to form a closed urethra. (D‐G) Create a hole in the deep fascia of the back and pass through the corpus cavernosum to place the new urethra in the abdomen of the penis

**FIGURE 7:**
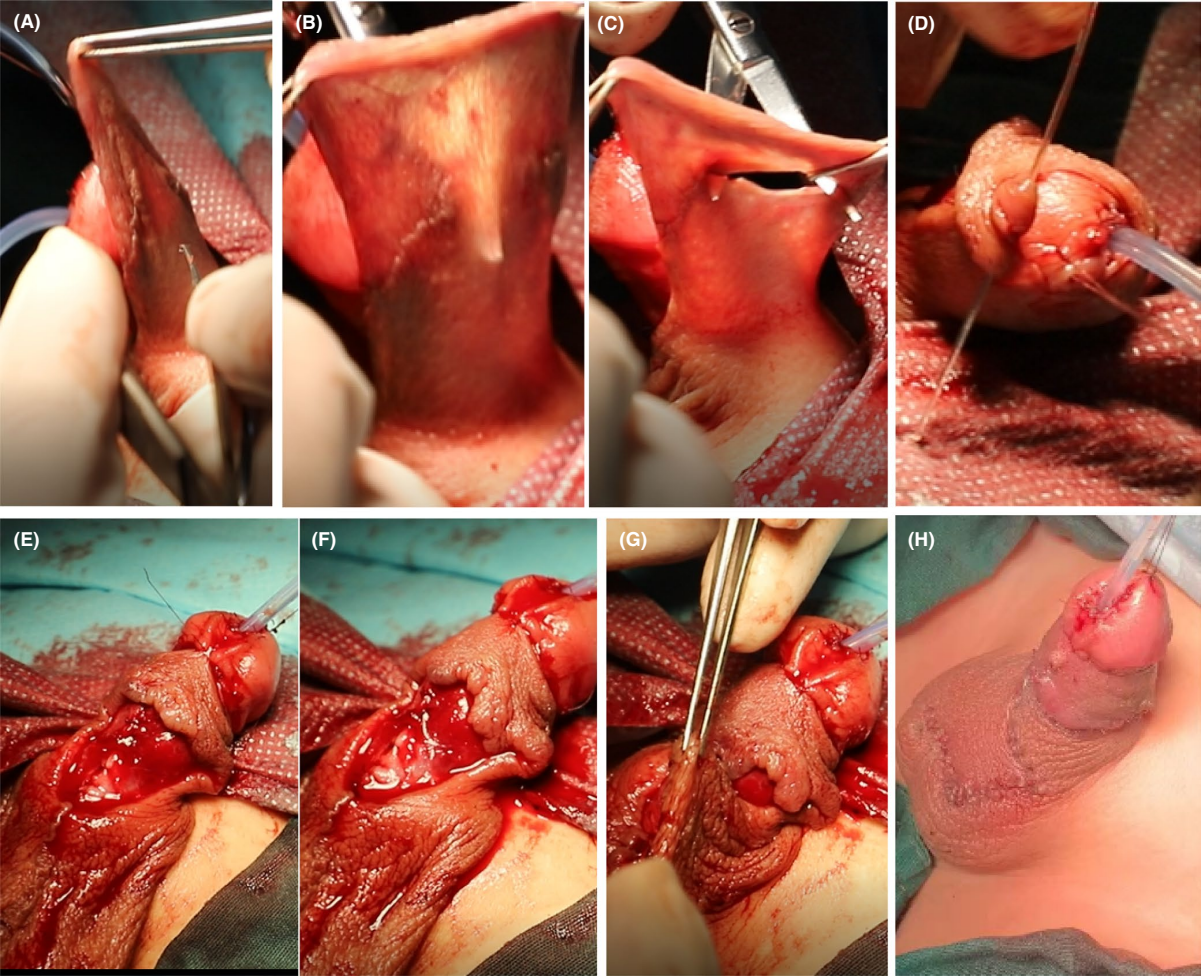
Locate the new urethral opening. (A‐C) Cut off the tip of the glans and create a tunnel in the abdomen of the glans. (D‐F) Pass the glans through the tunnel to locate the new urethral opening

**FIGURE 8:**
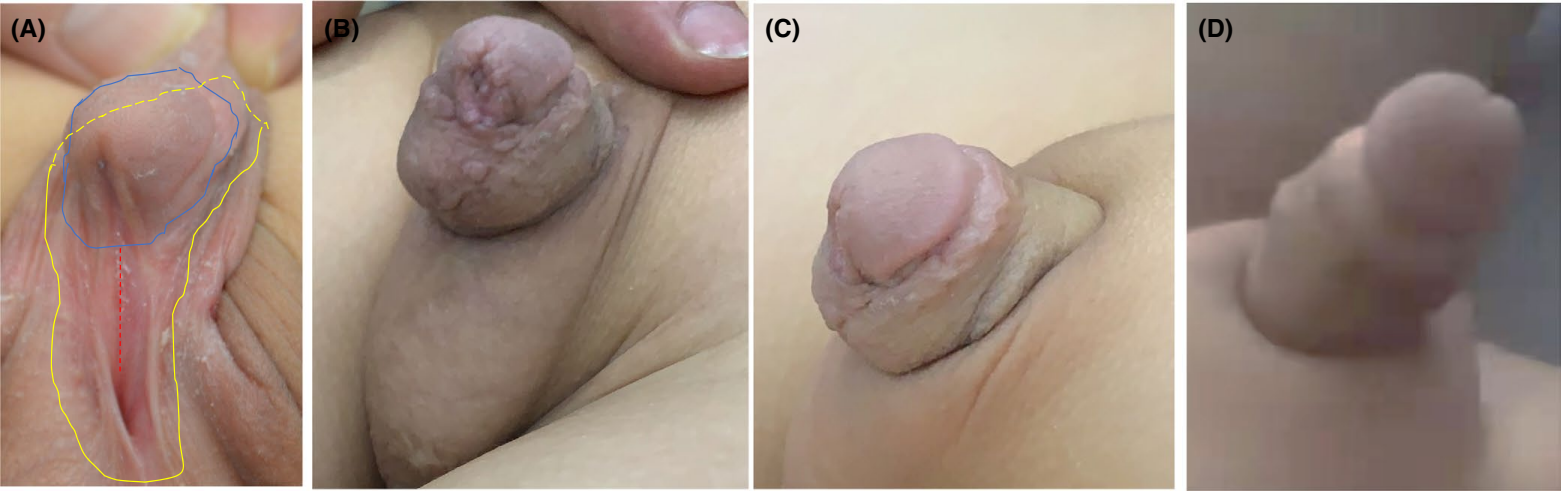
Suture the new urethral opening, and fix the urethra. (A‐C) Suture the new urethra. (D) Suture the new urethra

**FIGURE 9:**
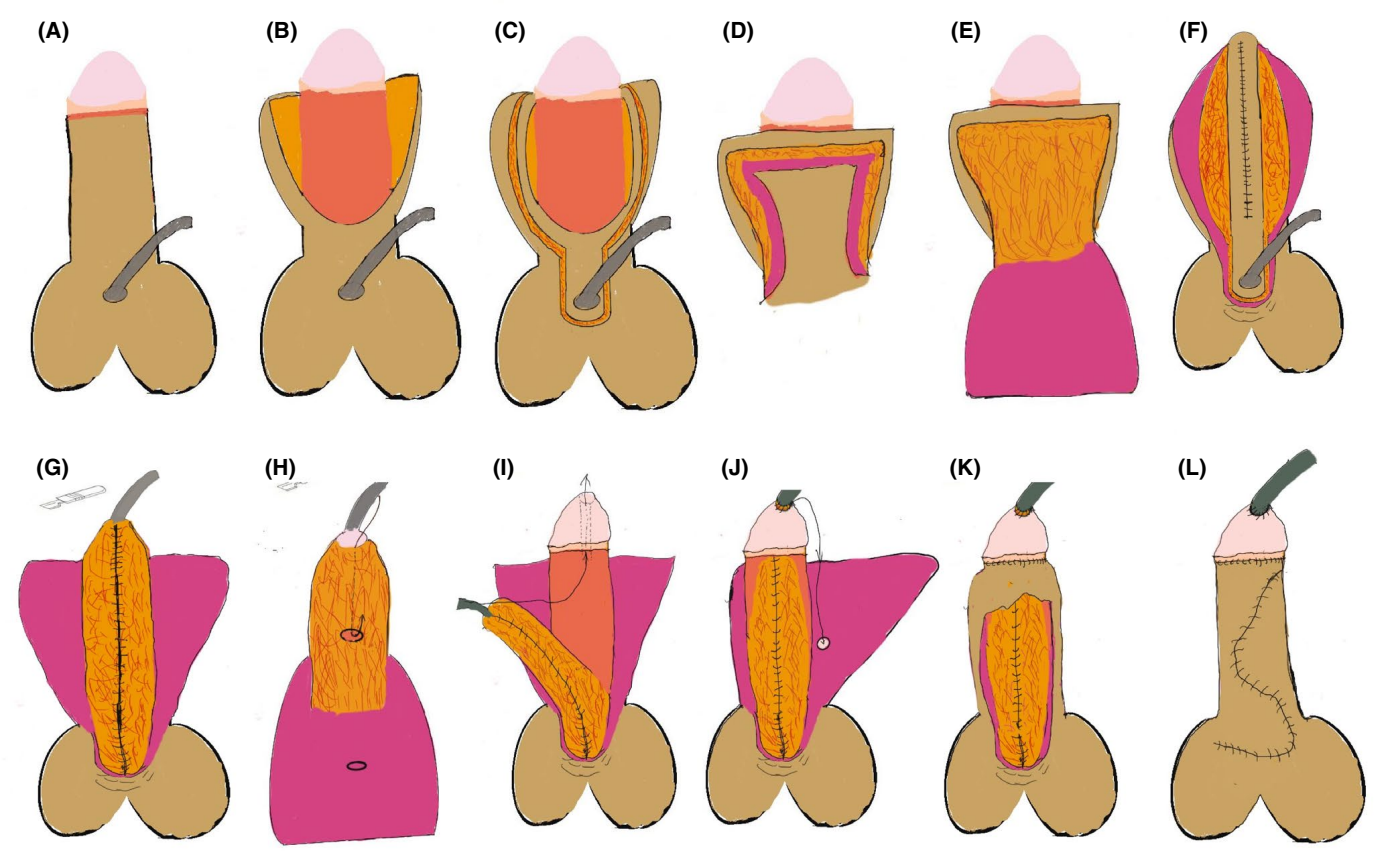
The coronary sulcus is located, and the outer skin of the penis is sutured. (A‐D) The remaining back skin and superficial fascia layer are punched and passed to form a new coronal foreskin. (E, F) Suture the foreskin and glans. (G, H) The remaining foreskin is sutured to form a complete penis

The surgical steps are as follows:

1. Circumcise the foreskin and separate the foreskin from the corpus cavernosum. A laser knife is used to circumcise the circumcision (cut off the deep fascia layer) along the dotted line, leaving the foreskin on the glans side 3–5 mm (Figure 1A‐B). The foreskin is separated from the corpus cavernosum albuginea along the corpus cavernosum (Figure 1C‐D; Figure 2A‐B). This step can straighten the curved cavernous body of the penis.
2. Create a 5‐mm‐wide, sealed, Y‐shaped skin flap. The skin and superficial fascia layer are incised along the incision 5 mm at the upper end of the foreskin to retain the deep fascia layer and the vascular layer above it, thus forming the upper part of the closed Y. At the same time, the skin and superficial fascia layer are incised at 5 mm on the left and right sides of the skin along the midline of the urethral outlet at 5 mm from the lower end of the urethral orifice to form the lower part of the Y‐line flap. This part is critical to the success of the operation. We need to carefully protect the vascular layer on the deep fascia layer from being damaged so that the blood supply of the flap used in the new urethra will not be necrotic (Figure 1A‐B; Figure 2C,D; Figure 3A‐I; Figure 4A).
3. Separate the dorsal foreskin along the superficial tendon layer: Use a scalpel to separate the foreskin into a superficial tendon layer and a deep tendon layer. Carefully use a scalpel to separate along the inner side of the superficial tendon layer without destroying the blood vessel layer. The skin will be divided into two layers: one layer is composed of a blood vessel layer and deep fascia, and the other layer is composed of skin and superficial fascia (Figure 2E; Figure 5A‐B).
4. Create a new urethra. The inner side of the Y‐shaped flap is sutured to form the inner side of the urethra (Figure 5C‐E), and then the urinary catheter is placed on it (Figure 6A). The outer side of the Y‐shaped flap is sutured to wrap the urinary catheter to form a new urethra. Here, the skin layer becomes the epithelium of the new urethra (Figure 2F,G; Figure 6B‐C).
5. Correct the position of the new urethra and penis: Create a hole in the middle of the deep tendon layer on the back (Figure 6D), then pass the sponge through this hole and straighten it (Figure 6E, F). In this way, the position of the new urethra and cavernous body is established (Figure 2H,I; Figure 6G).
6. Fix the urethral opening: Create a hole in the center of the glans on the ventral side of the penis, and cut off the top of the glans as a new urethral exit (Figure 7A‐C). The new urethra is passed through the glans (Figure 7D‐F), and then the urethral opening and glans incision are sutured (Figure 2J; Figure 8A‐C).
7. Fix the new urethra: Fix the urethra with absorbable sutures along the middle of the abdomen of the penis (Figure 8D)
8. Suture the coronary sulcus:Make a hole in the middle of the skin and superficial fascia layer separated in step 3 and then suture the urethral opening and the glans incision (Figure 9A‐C); next, pass the glans through the hole (Figure 9D). Suture the 3 mm foreskin left by step 1 and the opening to form a new coronal groove (Figure 2K; Figure 9E, F).
9. Suture the skin of the penis: Suture the remaining foreskin to form a complete skin (Figure 2L; Figure 9G‐H).

The penile wound was wrapped with petroleum jelly gauze, the scrotal wound was pressed with petroleum jelly, cefuroxime was used for 5 days after the operation, and the petroleum jelly‐impregnated gauze was removed 1 week after the operation. The urinary catheter was removed 4 weeks after the operation, and urination was observed.

Tips: (1) When constructing the Y‐shaped flap, only the superficial fascia layer can be cut, not the blood vessel layer.

(2) When separating the back foreskin, only the superficial fascia layer should be separated as much as possible, and the blood vessels between the superficial fascia layer and the deep fascia layer should not be injured. Not destroying blood vessels is the key to reducing complications and the success of this operation.

## 3 RESULTS

From 2014 to 2017, 29 cases of perineal hypospadias and 60 cases of scrotal hypospadias were performed at our institutions. The ages of the patients ranged from 1 to 10 years (mean = 4 years). All children had their urinary catheters removed 4 weeks after the operation. All children had their urinary catheters removed at 1, 3, and 6 months and then were evaluated at least once a year for 3 consecutive years. Nine cases of urine leakage occurred within 3 days after the operation. The leakage orifices were all located at the junction of the penis ventral penis and scrotum. The diameter of the leakage orifice was approximately 2–5 mm. One patient had urine leakage 3 months after the operation, and 1 patient had urine leakage 5 months after the operation. Urine leakage occurred in the first month, and the leakage opening was located in the middle of the ventral side of the penis, with a diameter of approximately 1 mm. It is believed that the needle eye caused the leakage of urine after the absorbable thread was absorbed. One case of urine leakage was caused by skin infection 1 year after the operation. All the children with urine leakage underwent repair after the leakage occurred 1 year later, and the leakage was successfully repaired at one time. There was no urethral stricture after the operation. The one‐time success rate of this operation was 87.6% (78/89), and the incidence of urethral fistula was 12.6% (11/89; Table S1).

## 4 DISCUSSION

The repair of proximal hypospadias is extremely challenging and has many surgical complications. At present, the more popular stage I surgery is as follows: The urethral plate is cut off to straighten the penis and thereby correct its curvature, and the urethra is formed by the cross‐cutting island‐shaped foreskin flap.^7^ Other modern techniques include Thiersch‐Duplay, onlay, and stage II surgery.^8^, ^9^ In 1975, Reddy first described urethral plate incision and tube urethroplasty.^10^ In 1994, the method of Snodgrass et al. was popularized, but recent long‐term follow‐up studies have shown that for proximal hypospadias, most children have functional urinary flow obstruction and decreased urinary flow rates after the operation.^11^ Since most children with proximal hypospadias have severe penile curvature, the fibrous tissue of the urethral plate needs to be removed for correction. Therefore, urethral plate preservation surgery (Thiersch‐Duplay, onlay, urethral plate incision, and roll tube urethroplasty) is not an ideal surgical method. In our approach, the foreskin of the coronal sulcus is used to form a closed Y‐shaped flap to extend the urethra. Our method not only repairs the damaged urethra but also completely corrects the curved penis (Figure 4A‐D).

Since 2000, more in‐depth research has been conducted on both sides of the base of the urethral orifice and the inner foreskin flap on the back to repair proximal hypospadias. Stage I urethroplasty with a continuously sealed Y skin flap of the penis and scrotum is a simple technique. Its advantages are as follows: (1) The material of the new urethral flap is sufficient. The new urethroplasty flap used in this operation is continuous from the urethral opening to the dorsal foreskin inner plate. The length of the flap is exactly the distance from the ectopic urethral orifice to the tip of the penis. The length of the flap should not be measured. (2) The original natural anatomical structure is preserved. It is currently believed that the urethral plate shows the natural urethroplastic structure of hypospadias, and the inner plate of the foreskin and the urethra come from the same germ layer during embryonic development. Utilizing the inner foreskin and urethral plates on both sides of the urethra orifice as the new urethroplasty material is most in line with the natural physiological anatomy of hypospadias; at the same time, the original axial blood supply of the entire skin flap of the new urethra is almost unchanged, which is conducive to wound healing and surgery. In this study, after the complications were repaired again, the success rate of the reoperation in this group was higher. (3) Bilateral scrotum sutures are used to eliminate scrotal division and translocation. (4) There is no anastomosis in the new urethra. The joint of the original urethral orifice of the operation is formed by a rolled tube urethral plate. There is no need for circular anastomosis, which can significantly reduce complications such as anastomotic stenosis. (5) The success rate is high. Preserving the blood supply of the distal foreskin so that the flaps will not be too long and receive a poor blood supply, especially with the scrotum or the testicular tunica on both sides of the scrotum covering the ventral side of the formed urethra to increase the level of tissue and prevent suture overlap, significantly reduces the occurrence of complications such as urinary fistula. (6) The surgical technique is not demanding, is easy to master, yields a good postoperative appearance, and facilitates smooth urination. In this group, 93.3% of the external genitalia were satisfactory in appearance, and the obstructive urinary flow curve was only 12.5%. Disadvantages: (1) There is a suture opening on the dorsal and ventral sides of the urethra, which needs to be sutured carefully, and the thread knot in the urethra, when sutured on the dorsal side, should not be too long. The author generally keeps 1–1.5 mm, and the absorption time of the absorbable thread should be within 1 month, otherwise the thread knot is too long and urination is not smooth; moreover, some of the thread‐knot urinary scale will condense, and urethral stones will appear. (2) The operation is simple, but it requires experience.

The indications for surgery are applicable to any severe proximal hypospadias, especially hypospadias accompanied by scrotal division and penile–scrotal translocation.

Intraoperative precautions: (1) The urethroplasty flap is a fascial flap pedicled at the urethral opening, and the blood supply of the flap must be protected; (2) When the new urethral flap is formed in a tubular shape, the thread knots are outside, with no tension, and the shape is formed. There is no leakage of urine afterward. (3) The anastomosis between the new urethral skin tube and the head of the penis is close to the ventral side, the new urethral opening will no longer be on the same side, and there will be no urethral stenosis. In short, stage I urethroplasty with a continuous Y skin flap of the penis and scrotum makes full use of and rarely destroys the natural anatomical and physiological structure of the original hypospadias. Thus, it is an ideal surgical method for proximal hypospadias.

## CONFLICT OF INTEREST

The authors declare no competing financial interests.

## AUTHOR CONTRIBUTIONS

JCZ and XWP designed the experiments JCZ, YFC, YLW, YL, and TQH analyzed the data. XWP and JCZ wrote the manuscript. JCZ, YFC, YL, TQH, YLW, YEZ, and XWP revised the manuscript. All authors approved the final version of the manuscript.

## ETHICAL APPROVAL

The Ethics Committee of Changsha Maternal and Child Health Hospital approved this study.

## CONSENT

Written consent has been obtained from the father/mother of the patient prior to the submission of this case report.

## Supporting information

Table S1

## Data Availability

All data, models, and code generated or used during the study appear in the submitted article.
